# Hospitalizations Among Active Component Members of the U.S. Armed Forces, 2024

**Published:** 2025-09-20

This report documents the frequencies, rates, trends, and distributions of hospitalizations among active component service members (ACSMs) of the U.S. Army, Navy, Air Force, Space Force, and Marine Corps during calendar year 2024. Summaries are based on standardized hospitalization records at U.S. military and non-military (reimbursed through the Military Health System) medical facilities worldwide that are routinely maintained in the Defense Medical Surveillance System (DMSS).

In this report, primary (i.e., first-listed) discharge diagnoses are considered indicative of the primary cause of hospitalization. As in prior *MSMR* reports, summaries are based on the first 3 digits of the International Classification of Diseases, 10th Revision (ICD-10) codes of the primary discharge diagnoses. Hospitalizations not routinely documented by standardized, automated records, e.g., during field training exercises or while shipboard, are not available in a centralized location for health surveillance purposes and are excluded from this report. Incidence rates were calculated per 1,000 person-years (p-yrs). Percent change in incidence was calculated using unrounded rates.

## Frequencies, rates and trends

In 2024, 58,860 hospitalizations were recorded for ACSMs of the U.S. Army, Navy, Air Force, Space Force, and Marine Corps **(Table 1)** ; 50.6% of these hospitalizations were in non-military facilities (data not shown), compared to 46.5% in 2023.

**TABLE 1.** Numbers, Rates ^a^ and Ranks ^b^ of Hospitalizations by ICD-10 Major Diagnostic Category, Active Component, U.S. Armed Forces, 2020, 2022 and 2024

	2020			2022			2024		
Major Diagnostic Category (ICD-10)	No.	Rate ^a^	Rank ^b^	No.	Rate ^a^	Rank ^b^	No.	Rate ^a^	Rank ^b^
Mental disorders (F01–F99)	18,284	13.8	1	21,525	16.6	1	17,212	13.8	1
Pregnancy and delivery (O00–O9A, relevant Z codes)	16,344	12.3	2	16,931	13.0	2	15,394	12.4	2
Pregnancy and delivery (O00–O9A, relevant Z codes) ^c^	16,344	71.74		16,931	74.61		15,394	69.25	
Injury, poisoning (S00–T88, DOD0101–DOD0105)	5,721	4.3	3	5,594	4.3	3	5,215	4.2	3
Digestive system (K00–K95)	5,280	4.0	4	4,991	3.8	4	4,665	3.8	4
Musculoskeletal system (M00–M99)	4,026	3.0	5	3,601	2.8	5	3,168	2.5	5
Signs, symptoms, ill-defined conditions (R00–R99)	2,465	1.9	6	2,410	1.9	6	2,152	1.7	6
Genitourinary system (N00–N99)	1,611	1.2	8	1,522	1.2	8	1,519	1.2	7
Respiratory system (J00–J99, U07.0)	1,516	1.1	10	1,291	1.0	11	1,516	1.2	8
Circulatory system (I00–I99)	1,520	1.1	9	1,503	1.2	9	1,456	1.2	9
Nervous system and sensory organ disorders (G00–G99, H00–H95)	1,293	1.0	11	1,338	1.0	10	1,348	1.1	10
Neoplasms (C00–D49)	1,232	0.9	12	1,223	0.9	12	1,181	0.9	11
Infectious and parasitic diseases (A00–B99)	884	0.7	13	921	0.7	13	1,130	0.9	12
Other (Z00–Z99, except pregnancy-related) ^d^	1,841	1.4	7	1,569	1.2	7	1,063	0.9	13
Skin and subcutaneous tissue (L00–L99)	752	0.6	14	703	0.5	14	783	0.6	14
Endocrine, nutritional, metabolic disorders (E00–E89)	575	0.4	15	511	0.4	15	528	0.4	15
Hematological and immune disorders (D50–D89)	295	0.2	17	272	0.2	17	249	0.2	16
Congenital anomalies (Q00–Q99)	208	0.2	18	210	0.2	18	224	0.2	17
COVID-19 (U07.1, U09.9)	527	0.4	16	291	0.2	16	57	0.05	18
Total	64,374	48.5		66,406	51.1		58,860	47.3	

Abbreviations: ICD, International Classification of Diseases, 10th Revision; No., number.

a Rate per 1,000 person-years.

b Rank of major diagnostic category based on number of hospitalizations.

c Rate of pregnancy and delivery-related hospitalizations among females only.

d Other factors influencing health status and contact with health services (excluding pregnancy-related).

**FIGURE 1.**
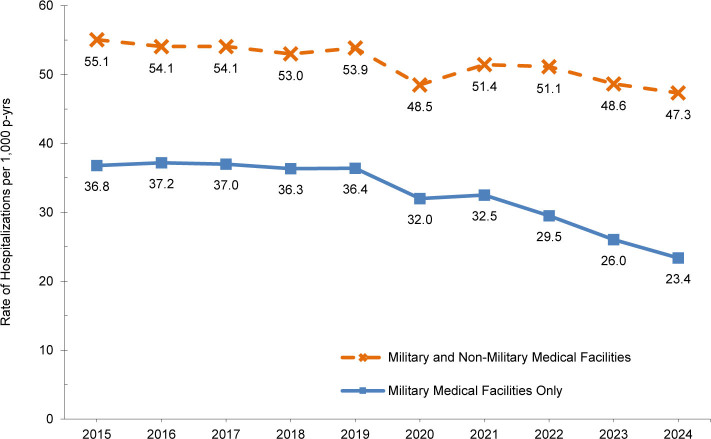
Rates of Hospitalization, by Type of Medical Facility, Active Component, U.S. Armed Forces, 2015–2024

Between 2015 and 2024, the total crude hospitalization rates declined gradually from a high of 55.1 per 1,000 p-yrs in 2015 to a low of 47.3 per 1,000 p-yrs in 2024, representing a decrease of 14% during the 10-year surveillance period. For military facilities, the decline was more pronounced, falling from 36.8 per 1,000 p-yrs in 2015 to 23.4 per 1,000 p-yrs in 2024, about a 36% reduction over the same period. The hospitalization rates between 2015 and 2019 were relatively stable, fluctuating within a narrow range. In 2020, an inflection point occurred, with rates dropping more than 10% below the 2019 level. Although rates rebounded near pre-COVID-19 pandemic levels in 2021 and 2022, they subsequently resumed a decline, reaching their lowest levels in 2024 **(Figure 1)** . Since 2020 was an atypical year due to COVID-19, causing disruptions in health care, this report mainly focuses on changes between 2022 and 2024.

## Hospitalizations, by ICD-10 major diagnostic categories

In 2024, just 4 ICD-10 major diagnostic categories accounted for almost three-quarters (72.2%) of all active component hospitalizations: mental health disorders (29.2%), pregnancy and delivery (26.2%), injury (8.9%), and digestive system (7.9%) **(Table 1)** . Consistent with findings for 2020 and 2022, hospitalizations for mental health disorders in 2024 accounted for more than any other major diagnostic category; 2009 was the last year in which any other diagnostic category—pregnancy and delivery—surpassed mental health disorder hospitalizations (data not shown).

The largest absolute reduction in hospitalizations occurred in the mental health disorders major diagnostic category, with 4,313 fewer hospitalizations in 2024 compared to 2022, translating into a 16.5% rate decrease **(Table 1)** . The number (rate decrease) of pregnancy and delivery hospitalizations decreased by 1,537 (-9.1%) cases, musculoskeletal conditions by 433 (-12.0%) cases, and the ‘other’ category by 506 (-32.2%) cases. The steepest rate drop, of nearly 80% (234 fewer cases), occurred in COVID-19 hospitalizations. Additional categories with comparatively large declines included injury (-379, -2.7%), digestive system (-326, -2.4%), and signs, symptoms and ill-defined conditions (-258, -6.8%), further contributing to the overall downward trajectory.

**What are the new findings?**

The hospitalization rate among U.S. active component service members in 2024 at both military and non-military medical facilities was 47.3 per 1,000 person-years, the lowest since 2015, continuing the general declining trend observed over the previous 9 years. It also represents a reduction of 14.0% from the 2015 peak, and 2.8% from the 2023 rate. As in prior years, over half (55.4%) of hospitalizations for active component members were associated with primary diagnoses in 2 categories: mental health disorders and pregnancy conditions.

**What is the impact on readiness and force health protection?**

As in prior years, mental health disorders, including substance abuse disorders, were associated with the longest median hospital stay, 6 days; 5% of hospitalizations for mental health disorders had durations greater than 30 days. Prolonged hospitalizations, after care, and early attrition due to these common disorders can diminish not merely individual but unit operational readiness.

At the same time, several major diagnostic categories increased in both frequency and rate of hospitalizations. The largest increases were observed in the respiratory system (225 additional cases, 22.6% rate increase), infectious and parasitic diseases (209, 28.1%), and skin and subcutaneous tissue (80, 16.3%) diagnostic categories.

## Hospitalizations, by sex

The hospitalization rate (for all causes) for active component service women in 2024 was more than 3 times that of service men (112.2 per 1,000 p-yrs vs. 33.2 per 1,000 p-yrs, respectively). These data are consistent with hospitalization rate trends published in 2022 for women and men ages 18-44 years (95 per 1,000 p-yrs and 37 per 1,000 p-yrs, respectively) in the general U.S. population. ^1^ Excluding pregnancy and delivery, the rate of hospitalizations among women (42.2 per 1,000 p-yrs) was 29.2% higher than among men (33.2 per 1,000 p-yrs) in 2024 (data not shown). This rate difference was primarily due to hospitalizations for mental health disorders (female:male rate difference [RD] 4.8 per 1,000 p-yrs) and genitourinary systems (RD 2.3 per 1,000 p-yrs) (data not shown).

**FIGURE 2.**
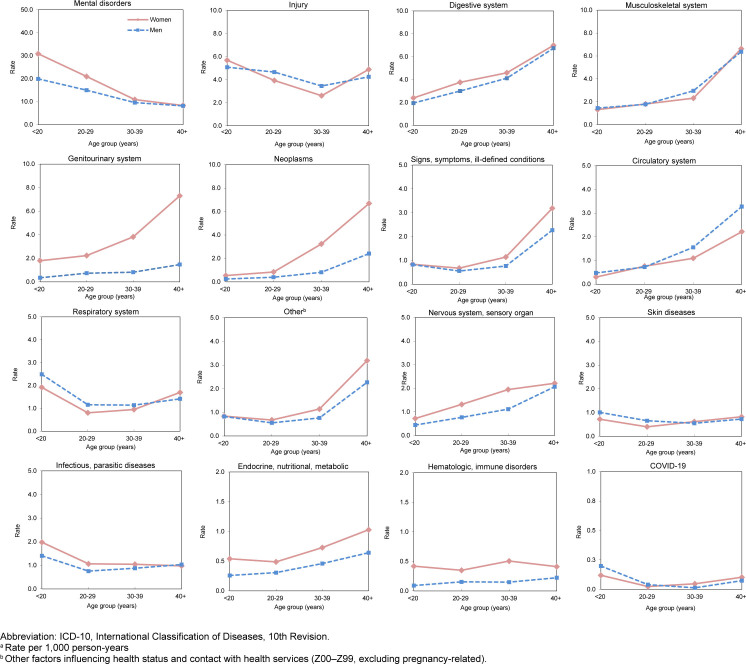
Rates ^a^ of Hospitalization by ICD-10 Major Diagnostic Category, Age Group and Sex, Active Component, U.S. Armed Forces, 2024

Relationships between age and hospitalization rates varied by major diagnostic category **(Figure 2)** . Rates among women in all age groups were consistently higher for the mental health disorders, genitourinary system, nervous and sensory organ diseases, digestive systems, neoplasms, endocrine, nutritional, metabolic, hematological, and immune disorders, and the ‘other’ diagnostic category. As in prior years, the sex gap was greatest for conditions in genitourinary system category, with females admitted at rates 3 to 5 times those of males of all age groups. Similarly, hospitalizations rates for neoplasms, hematological and immune disorders were more than twice as high among women compared to men. In contrast, rates among men were higher than those among women in all age groups for conditions in the respiratory system category. Hospitalization rates of mental health disorders were 50% higher among younger women, under age 30 years, and were comparable among older age groups.

Hospitalization rates among both sexes generally increased with age for most diagnostic categories except mental health disorders, injury, skin and subcutaneous tissue, respiratory system, infectious and parasitic diseases, and COVID-19. Rates decreased for both sexes with increasing age for mental health disorders and were relatively stable among all age groups for infectious and parasitic diseases, skin and subcutaneous tissue categories, and hematological and immune disorders.

## Most frequent diagnoses

Mental health disorders represented a significant portion of hospital admissions among ACSMs. Mental health disorder diagnoses, collectively, accounted for over 40% of all hospitalizations among men and women—excluding pregnancy and delivery. Adjustment disorders were the primary discharge diagnosis for both men (n=3,869) and women (n=1,127) **(Tables 2** and **3)** in 2024, accounting for nearly 30% of total mental health disorder hospitalizations. The next 4 most frequent mental health diagnoses, for both sexes, were alcohol- and depression-related disorders, including recurrent major depressive disorder (severe without psychotic features), and post-traumatic stress disorder (PTSD). The pregnancy and delivery category constituted the top major diagnostic category for women, accounting for over three-fifths (62.6%) of all female hospitalizations **(Table 3)** .

**TABLE 2.** Numbers and Percentages of the Most Frequent Diagnoses During Hospitalization Among Men ♂, by ICD-10 Major Diagnostic Category, Active Component, U.S. Armed Forces, 2024

Diagnostic Category (ICD-10 codes)	No.	% ^a^
**Mental disorders (F01-F99)**	**13,267**	
Adjustment disorder	3,869	29.2
Alcohol dependence	2,565	19.3
Major depressive disorder, recurrent severe without psychotic features	1,355	10.2
Post-traumatic stress disorder (PTSD)	582	4.4
Depression, unspecified	502	3.8
**Injury, poisoning (S00–T98, DOD0101–DOD0105)**	**4,376**	
Concussion	200	4.6
Infection following procedure	161	3.7
Fracture of shaft of tibia	122	2.8
Other fractures of lower leg	99	2.3
Fracture of shaft of femur	98	2.2
**Digestive system (K00–K95)**	**3,737**	
Other and unspecified acute appendicitis	814	21.8
Acute appendicitis with localized peritonitis	250	6.7
Acute pancreatitis, unspecified	165	4.4
Alcohol induced acute pancreatitis	147	3.9
Calculus of gallbladder with acute cholecystitis	116	3.1
**Musculoskeletal system (M00–M99)**	**2,649**	
Other specified disorders of muscle	467	17.6
Thoracic, thoracolumbar, lumbosacral intervertebral disc disorders with radiculopathy	212	8.0
Spinal stenosis	182	6.9
Anomalies of dental arch relationship	148	5.6
Other spondylosis with radiculopathy	101	3.8
**Symptoms, signs, abnormal clinical and laboratory findings, NEC (R00–R99)**	**1,667**	
Other symptoms and signs involving emotional state	345	20.7
Syncope and collapse	153	9.2
Other symptoms and signs involving cognitive functions and awareness	128	7.7
Chest pain, unspecified	95	5.7
Other chest pain	79	4.7
**Circulatory system (I00–I99)**	**1,246**	
Pulmonary embolism without acute cor pulmonale	124	10.0
Non-ST elevation (NSTEMI) myocardial infarction	77	6.2
Paroxysmal atrial fibrillation	58	4.7
Unspecified atrial fibrillation and atrial flutter	48	3.9
Atherosclerotic heart disease of native coronary artery	38	3.0
**Respiratory system (J00-J99, U07.0)**	**1,292**	
Pneumonia, unspecified organism	211	16.3
Peritonsillar abscess	110	8.5
Deviated nasal septum	81	6.3
Acute respiratory failure	73	5.7
Other pneumothorax and air leak	66	5.1
**Nervous system and sensory organ disorders (G00–G99, H00–H95)**	**1,008**	
Sleep apnea	100	9.9
Epilepsy, unspecified	56	5.6
Acute pain, not elsewhere classified	31	3.1
Transient cerebral ischemic attack, unspecified	30	3.0
Brachial plexus disorder	28	2.8
** Other (Z00–Z99, except pregnancy-related) ^b^**	**832**	
Encounter for antineoplastic chemotherapy and immunotherapy	182	21.9
Aftercare following joint replacement surgery	141	16.9
Encounter for other orthopedic aftercare	102	12.3
Encounter for other specified post-procedural aftercare	53	6.4
Encounter for examination and observation for unspecified reason	45	5.4
**Infectious and parasitic diseases (A00–B99)**	**881**	
Sepsis, unspecified organism	413	46.9
Infectious gastroenteritis, colitis, unspecified	71	8.1
Other specified sepsis	33	3.7
Viral intestinal infection, unspecified	24	2.7
Sepsis due to other Gram-negative organisms	22	2.5
**Genitourinary system (N00–N99)**	**831**	
Acute kidney failure, unspecified	223	26.8
Hydronephrosis with renal and ureteral calculous obstruction	75	9.0
Calculus of ureter	43	5.2
Torsion of testis	37	4.5
Calculus of kidney	31	3.7
**Neoplasms (C00–D49)**	**735**	
Malignant neoplasm of thyroid gland	28	3.8
Malignant neoplasm of rectum	24	3.3
Acute myeloblastic leukemia	23	3.1
Malignant neoplasm of prostate	21	2.9
Benign neoplasm of pituitary gland	21	2.9
**Skin and subcutaneous tissue (L00–L99)**	**667**	
Cellulitis and acute lymphangitis of other parts of limb	283	42.4
Cellulitis and acute lymphangitis of face, neck	50	7.5
Cutaneous abscess, furuncle and carbuncle of limb	35	5.2
Pilonidal cyst and sinus with abscess	32	4.8
Cellulitis and acute lymphangitis of finger, toe	26	3.9
**Endocrine, nutritional, metabolic diseases (E00–E89)**	**393**	
Type 2 diabetes mellitus with ketoacidosis	57	14.5
Type 1 diabetes mellitus with ketoacidosis	49	12.5
Dehydration	39	9.9
Type 2 diabetes mellitus with other specified complications	34	8.7
Hypo-osmolality, hyponatremia	24	6.1
**Hematological and immune disorders (D50–D89)**	**159**	
Neutropenia, unspecified	18	11.3
Immune thrombocytopenic purpura	17	10.7
Other specified aplastic anemias and other bone marrow failure syndromes	12	7.5
Acute posthemorrhagic anemia	11	6.9
Agranulocytosis secondary to cancer chemotherapy	11	6.9
**Congenital anomalies (Q00–Q99)**	**139**	
Other congenital deformities of hip	15	10.8
Atrial septal defect	14	10.1
Arteriovenous malformation of cerebral vessels	12	8.6
Malformation of coronary vessels	8	5.8
Meckel's diverticulum (displaced) (hypertrophic)	7	5.0
**COVID-19 (ICD-10: U07.1, U09.9)**	**47**	
COVID-19	47	100

Abbreviations: ICD, International Classification of Diseases, 10th Revision; No., number; NSTEMI, non-ST segment elevation myocardial infarction; NEC, not elsewhere classified.

a Percentage of the total number of hospitalizations within the diagnostic category.

b Other factors influencing health status and contact with health services (excluding pregnancy-related).

**TABLE 3.** Numbers and Percentages of the Most Frequent Diagnoses During Hospitalization Among Women ♀, by ICD-10 Major Diagnostic Category, Active Component, U.S. Armed Forces, 2024

Diagnostic Category (ICD-10-CM codes)	No.	% ^a^
**Pregnancy and delivery (O00–O99, relevant Z codes)**	**15,394**	
Post-term pregnancy	1,621	10.5
Maternal care due to uterine scar from previous surgery	949	6.2
Abnormality in fetal heart rate, rhythm complicating labor and delivery	835	5.4
Premature rupture of membranes, onset of labor within 24 hours of rupture	833	5.4
Gestational [pregnancy-induced] hypertension without significant proteinuria, complicating childbirth	749	4.9
**Mental disorders (F01–F99)**	**3,945**	
Adjustment disorder	1,127	28.6
Major depressive disorder, recurrent severe without psychotic features	531	13.5
Post-traumatic stress disorder (PTSD)	386	9.8
Alcohol dependence	307	7.8
Depression, unspecified	158	4.0
**Digestive system (K00–K95)**	**928**	
Other and unspecified acute appendicitis	167	18.0
Calculus of gallbladder with acute cholecystitis	69	7.4
Calculus of gallbladder with other cholecystitis	48	5.2
Acute cholecystitis	42	4.5
Acute appendicitis with localized peritonitis	29	3.1
**Injury, poisoning (S00–T98, DOD0101–DOD0105)**	**839**	
Poisoning by, adverse effect of, under-dosing of 4-aminophenol derivatives	53	6.3
Infection following a procedure	47	5.6
Poisoning by, adverse effect of, under-dosing of other and unspecified antidepressants	45	5.4
Other fractures of lower leg	26	3.1
Unspecified injury	25	3.0
**Genitourinary system (N00–N99)**	**688**	
Abnormal uterine and vaginal bleeding, unspecified	116	16.9
Acute pyelonephritis	51	7.4
Other and unspecified ovarian cysts	47	6.8
Hypertrophy of breast	46	6.7
Tubulo-interstitial nephritis, not specified as acute or chronic	24	3.5
**Musculoskeletal system (M00–M99)**	**519**	
Anomalies of dental arch relationship	49	9.4
Other specified disorders of muscle	35	6.7
Spinal stenosis	30	5.8
Major anomaly of jaw size	29	5.6
Stress fracture	28	5.4
**Symptoms, signs, abnormal clinical and laboratory findings, NEC (R00–R99)**	**485**	
Other symptoms and signs involving emotional state	93	19.2
Syncope and collapse	49	10.1
Unspecified abdominal pain	42	8.7
Pain localized to other parts of lower abdomen	28	5.8
Pain localized to upper abdomen	22	4.5
**Neoplasms (C00–D49)**	**446**	
Leiomyoma of uterus, unspecified	116	26.0
Intramural leiomyoma of uterus	59	13.2
Subserosal leiomyoma of uterus	38	8.5
Malignant neoplasm of breast of unspecified site	23	5.2
Malignant neoplasm of thyroid gland	14	3.1
**Nervous system and sensory organ disorders (G00–G99, H00–H95)**	**340**	
Acute pain, not elsewhere classified	30	8.8
Migraine, unspecified	18	5.3
Brachial plexus disorders	17	5.0
Migraine with aura	16	4.7
Benign intracranial hypertension	16	4.7
** Other (Z00–Z99, except pregnancy-related) ^b^**	**231**	
Encounter for other orthopedic aftercare	41	17.7
Aftercare following joint replacement surgery	30	13.0
Encounter for examination and observation for unspecified reason	24	10.4
Encounter for other specified post-procedural aftercare	16	6.9
Encounter for breast reconstruction following mastectomy	14	6.1
**Infectious and parasitic diseases (A00–B99)**	**249**	
Sepsis, unspecified organism	114	45.8
Sepsis due to other Gram-negative organisms	22	8.8
Infectious gastroenteritis and colitis, unspecified	20	8.0
Other specified sepsis	9	3.6
Viral intestinal infection, unspecified	6	2.4
**Circulatory system (I00–I99)**	**210**	
Pulmonary embolism without acute cor pulmonale	33	15.7
Supraventricular tachycardia	13	6.2
Acute embolism and thrombosis of deep veins of lower extremity	13	6.2
Essential (primary) hypertension	7	3.3
Non-ST elevation (NSTEMI) myocardial infarction	7	3.3
**Respiratory system (J00–J99, U07.0)**	**224**	
Pneumonia, unspecified organism	36	16.1
Acute respiratory failure	15	6.7
Peritonsillar abscess	13	5.8
Other intraoperative, post-procedural complications and disorders of respiratory system, not elsewhere classified	13	5.8
Acute tonsillitis, unspecified	12	5.4
**Endocrine, nutritional, metabolic diseases (E00–E89)**	**135**	
Thyrotoxicosis with diffuse goiter	18	13.3
Type 2 diabetes mellitus with ketoacidosis	11	8.1
Dehydration	10	7.4
Hypokalemia	9	6.7
Obesity due to excess calories	7	5.2
**Skin and subcutaneous tissue (L00–L99)**	**116**	
Cellulitis and acute lymphangitis of other parts of limb	33	28.4
Pilonidal cyst and sinus with abscess	8	6.9
Cellulitis and acute lymphangitis of face, neck	7	6.0
Cutaneous abscess, furuncle and carbuncle of limb	6	5.2
Cellulitis and acute lymphangitis of finger, toe	6	5.2
**Congenital anomalies (Q00–Q99)**	**85**	
Iron deficiency anemia, unspecified	21	23.3
Acute posthemorrhagic anemia	15	16.7
Iron deficiency anemia secondary to blood loss (chronic)	9	10.0
Anemia, unspecified	7	7.8
Immune thrombocytopenic purpura	7	7.8
**Hematological and immune disorders (D50–D89)**	**90**	
Anemia, unspecified	19	21.1
Iron deficiency anemia, unspecified	14	15.6
Iron deficiency anemia secondary to blood loss (chronic)	10	11.1
Other iron deficiency anemias	7	7.8
Acute posthemorrhagic anemia	7	7.8
**COVID-19 (U07.1, U09.9)**	**10**	
COVID-19	9	90.0
Post COVID-19 condition, unspecified	1	10.0

Abbreviations: ICD, International Classification of Diseases, 10th Revision; No., number; NSTEMI, non-ST segment elevation myocardial infarction; NEC, not elsewhere classified.

a Percentage of the total number of hospitalizations within diagnostic category.

b Other factors influencing health status and contact with health services (excluding pregnancy-related).

Other common causes of hospitalization, regardless of sex, included other and unspecified acute appendicitis; sepsis, unspecified organism; and other symptoms and signs involving emotional state; as well as other specified disorders of muscle for men and abnormal uterine and vaginal bleeding for women.

## Durations of hospitalizations

**FIGURE 3.**
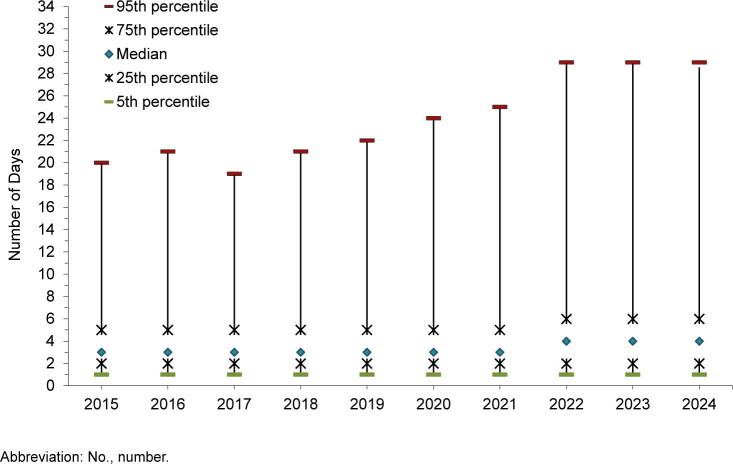
Duration of Hospital Stay, Active Component, U.S. Armed Forces, 2015–2024

When graphically represented, hospitalization durations demonstrate a highly right-skewed (positive) distribution, with the lower limit equal to 1 day and a mode of 3 days. Because length of hospital stay is not normally distributed, the median duration with interquartile range (IQR) was chosen as the best measure of central tendency. The median (IQR) duration of hospital stays (for all causes) has remained generally stable at 3 (2-5) days but increased to 4 (2-6) days in 2022 and has remained at that level **(Figure 3)** .

Median duration days of hospitalization varied substantially by major diagnostic category. The shortest durations of stays (median days, IQR) were observed for musculoskeletal system, genitourinary system, and digestive system hospitalizations (2 days, 2-6). The longest stays were for mental health disorder (6 days, 4-13) and ‘other’ (5 days, 3-16) hospitalizations. The remaining categories had a median of 3 (2-7) days.

**FIGURE 4.**
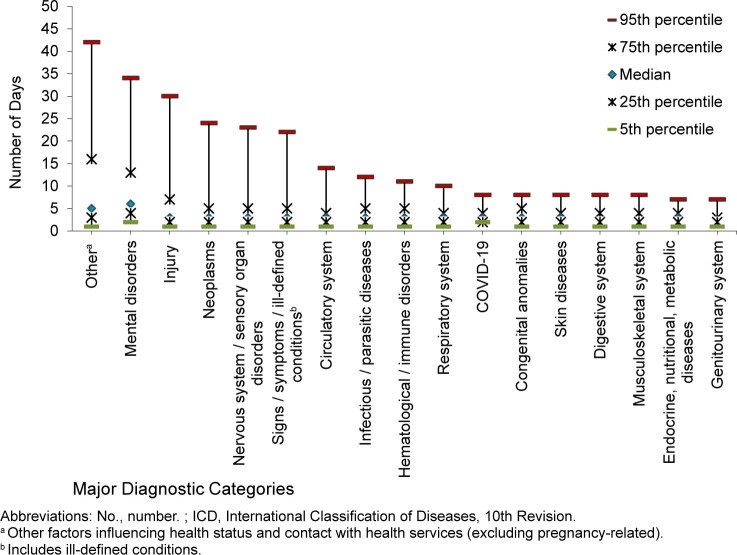
Duration of Hospital Stay by ICD-10 Major Diagnostic Category, Active Component, U.S. Armed Forces, 2015–2024

Five percent of hospitalization stays exceeded 10 days for one half of ICD diagnostic categories: hematological and immune disorders (11 days), infectious and parasitic diseases (12 days), circulatory system (14 days), signs, symptoms and ill-defined conditions (22 days), nervous system and sensory organ diseases (23 days), neoplasms (24 days), injury (30 days), mental health disorders (34 days), and ‘other’ (primarily orthopedic aftercare and rehabilitation following prior illness or injury) (42 days) **(Figure 4)** .

## Hospitalizations, by service

Among active component members of the Navy, Air Force, and Space Force, pregnancy and delivery accounted for more hospitalizations than any other diagnostic category, while among ACSMs of the Army and Marine Corps, mental health disorders were the leading cause of hospitalization **(Table 4)** . Prior to 2020, pregnancy and delivery were ranked first for both Navy and Air Force ACSMs. Among all the services, the crude hospitalization rate for mental health disorders in 2024 was highest among Army ACSMs (16.1 per 1,000 p-yrs).

**TABLE 4.** Numbers and Rates ^a^ of Hospitalizations, by Service and ICD-10 Diagnostic Category, Active Component, U.S. Armed Forces, 2024

	Army		Navy		Air Force		Space Force		Marine Corps	
Major Diagnostic Category (ICD-10 codes)	No.	Rate ^a^	No.	Rate ^a^	No.	Rate ^a^	No.	Rate ^a^	No.	Rate ^a^
Mental disorders (F01–F99)	7,048	16.1	4,338	13.4	3,276	10.6	66	7.2	2,484	15.0
Pregnancy and delivery (O00–O9A, relevant Z codes)	5,339	12.2	4,486	13.9	4,220	13.7	95	10.3	1,254	7.6
Injury, poisoning (S00–T88, DOD0101–DOD0105)	2,365	5.4	1,151	3.6	857	2.8	11	1.2	831	5.0
Digestive system (K00–K95)	1,964	4.5	1,231	3.8	927	3.0	26	2.8	517	3.1
Musculoskeletal system (M00–M99)	1,565	3.6	619	1.9	572	1.9	19	2.1	393	2.4
Signs, symptoms, ill-defined conditions (R00–R99)	1,080	2.5	482	1.5	373	1.2	16	1.7	201	1.2
Genitourinary system (N00–N99)	654	1.5	339	1.1	374	1.2	8	0.9	144	0.9
Respiratory system (J00–J99, U07.0)	631	1.4	335	1.0	294	1.0	9	1.0	247	1.5
Circulatory system (I00–I99)	623	1.4	364	1.1	326	1.1	7	0.8	136	0.8
Nervous system and sensory organ disorders (G00–G99, H00–H95)	621	1.4	319	1.0	264	0.9	7	0.8	137	0.8
Neoplasms (C00–D49)	447	1.0	294	0.9	341	1.1	14	1.5	85	0.5
Infectious and parasitic diseases (A00–B99)	429	1.0	296	0.9	238	0.8	9	1.0	158	1.0
Other (Z00–Z99, except pregnancy-related) ^b^	426	1.0	245	0.8	239	0.8	10	1.1	143	0.9
Skin and subcutaneous tissue (L00–L99)	336	0.8	191	0.6	96	0.3	5	0.5	155	0.9
Endocrine, nutritional, metabolic diseases (E00–E89)	233	0.5	149	0.5	90	0.3	4	0.4	52	0.3
Hematological and immune disorders (D50–D89)	105	0.2	52	0.2	72	0.2	2	0.2	18	0.1
Congenital anomalies (Q00–Q99)	86	0.2	56	0.2	42	0.1			40	0.2
COVID-19 (U07.1, U09.9)	20	0.0	13	0.0	13	0.0	2	0.2	9	0.1
Total	23,972	54.8	14,960	46.4	12,614	40.8	310	33.6	7,004	42.3

Abbreviations: ICD, International Classification of Diseases; No., number.

a Rates are based on 1,000 person-years.

b Other factors influencing health status and contact with health services (excluding pregnancy-related).

Injury was the third leading hospitalization category among Army and Marine Corps ACSMs, 5.4 per 1,000 p-yrs and 5.0 per 1,000 p-yrs, respectively. Among Navy, Air Force and Space Force ACSMs, the third highest rate of hospitalizations was for the digestive system category, at 3.8, 3.0, and 2.8 per 1,000 p-yrs, respectively.

## Discussion

The 2024 crude annual hospitalization rate marks the lowest recorded level since 2015, continuing a general downward trend observed over the last 10 years. The decline appears largely driven by reductions in hospitalizations in mental health disorders, pregnancy and delivery, musculoskeletal system, and ‘other’ categories. A significant decrease in hospitalizations in 2020 coincided with COVID-19 pandemic-related changes in health care provision, while the post-pandemic period saw a dramatic drop in COVID-19 hospitalizations.

As in past years, in 2024 mental health disorders accounted for more hospitalizations than any other major diagnostic category. Within the mental health disorders category, adjustment disorders, alcohol dependence, depressive disorders, and PTSD were among the leading primary discharge diagnoses for both men and women. At the same time, modest increases were observed in both hospitalization frequencies and rates for respiratory system, infectious and parasitic diseases, and skin and subcutaneous tissue categories. Neoplasms and circulatory system categories demonstrated small absolute declines but slight rate increases, likely due to denominator (person-time) or demographic fluctuations. Although the overall hospitalization rate continued to decline in 2024, these findings indicate that the downward trend was not uniform for diagnostic categories.

Certain limitations should be considered when interpreting these results. This summary is based on primary (first-listed) discharge diagnoses only, but in many hospitalized cases, multiple conditions can be present; for example, joint pain (category, musculoskeletal) may be co-listed with an injury (category, injury). In such cases, only the first-listed discharge diagnosis would be accounted in this report. Discharge coding among multiple categories could lead to under-estimation of hospitalization rates for common conditions.

Since May 2023, DMSS data have been housed and analyzed from the Military Health System Information Platform (MIP). All military treatment facilities are now using GENESIS software to electronically capture medical care. Data completeness issues related to data transfers from GENESIS to the Medical Data Store (MDR) to DMSS have improved significantly. Regardless of the electronic system used to capture hospitalizations, every hospitalization record requires completion of a discharge summary before the event record is reported in the system. Consequently, timeliness of reporting can still be an issue that may lead to under-estimates of true counts and rates of hospitalizations for the most recent year of reporting. As a result, direct comparison between the 2024 data and data from prior years should be interpreted with caution.
